# Genetic and epigenetic mechanisms influencing acute to chronic postsurgical pain transitions in pediatrics: Preclinical to clinical evidence

**DOI:** 10.1080/24740527.2021.2021799

**Published:** 2022-05-10

**Authors:** Adam J. Dourson, Adam Willits, Namrata G.R. Raut, Leena Kader, Erin Young, Michael P. Jankowski, Vidya Chidambaran

**Affiliations:** a Department of Anesthesia, Division of Pain Management, Cincinnati Children’s Hospital Medical Center, Cincinnati, Ohio,USA; b Neuroscience Graduate Program, University of Kansas Medical Center, Kansas City, Kansas, USA; c Department of Anatomy and Cell Biology, University of Kansas Medical Center, Kansas City, Kansas, USA; d Department of Anesthesiology, University of Kansas Medical Center, Kansas City, Kansas, USA; e Department of Pediatrics, University of Cincinnati, College of Medicine, Cincinnati, Ohio, USA

**Keywords:** chronic postsurgical pain, variants, association studies, GWAS, DNA methylation, epigenetics, immunogenetics, nociception, rodent models, clinical, CPSP

## Abstract

**Background:**

Chronic postsurgical pain (CPSP) in children remains an important problem with no effective preventive or therapeutic strategies. Recently, genomic underpinnings explaining additional interindividual risk beyond psychological factors have been proposed.

**Aims:**

We present a comprehensive review of current preclinical and clinical evidence for genetic and epigenetic mechanisms relevant to pediatric CPSP.

**Methods:**

Narrative review.

**Results:**

Animal models are relevant to translational research for unraveling genomic mechanisms. For example, *Cacng2, p2rx7*, and *bdnf* mutant mice show altered mechanical hypersensitivity to injury, and variants of the same genes have been associated with CPSP susceptibility in humans; similarly, differential DNA methylation (*H1SP*) and miRNAs (miR-96/7a) have shown translational implications. Animal studies also suggest that crosstalk between neurons and immune cells may be involved in nociceptive priming observed in neonates. In children, differential DNA methylation in regulatory genomic regions enriching GABAergic, dopaminergic, and immune pathways, as well as polygenic risk scores for enhanced prediction of CPSP, have been described. Genome-wide studies in pediatric CPSP are scarce, but pathways identified by adult gene association studies point to potential common mechanisms.

**Conclusions:**

Bench-to-bedside genomics research in pediatric CPSP is currently limited. Reverse translational approaches, use of other -omics, and inclusion of pediatric/CPSP endophenotypes in large-scale biobanks may be potential solutions. Time of developmental vulnerability and longitudinal genomic changes after surgery warrant further investigation. Emergence of promising precision pain management strategies based on gene editing and epigenetic programing emphasize need for further research in pediatric CPSP-related genomics.

## Abstract (fr)

**Contexte:**

La douleur chronique post-chirurgicale (DCPC) chez l'enfant reste un problème important pour lequel il n’y a pas de stratégies préventives ou thérapeutiques efficaces. Récemment, des fondements génomiques expliquant des risques interindividuels additionnels, au-delà des facteurs psychologiques, ont été proposés.

**Objectifs:**

Nous présentons une revue compléte des données probantes précliniques et cliniques actuelles pour les mécanismes génétiques et épigénétiques pertinents en matiére de DCPC pédiatrique.

**Méthodes:**

Revue narrative.

**Résultats:**

Les modéles animaux sont pertinents pour la recherche translationnelle afin de déchiffrer les mécanismes génomiques. Par exemple, les souris mutantes Cacng2, p2rx7 et bdnf présentent une hypersensibilité mécanique altérée à des lésions et des variantes des mêmes génes ont été associées à la sensibilité à la DCPC chez l’humain; de même, la méthylation différentielle de l'ADN (H1SP) et les miARN (miR-96/7a) ont montré des implications translationnelles. Des études menées sur des animaux indiquent également que la diaphonie entre les neurones et les cellules immunitaires peut être impliquée dans l'amorçage nociceptif observé chez les nouveau-nés. Chez les enfants, la méthylation différentielle de l'ADN dans les régions génomiques régulatrices enrichissant les voies GABAergiques, dopaminergiques et immunitaires, ainsi que des scores de risque polygénique pour une prédiction améliorée de la PCSP, ont été décrits. Les études pangénomiques en matiére de DCPC pédiatrique sont rares, mais les voies identifiées par les études d'association de génes chez l'adulte indiquent de possibles mécanismes communs.

**Conclusions:**

La recherche en génomique du laboratoire au patient dans le cadre de la DCPC pédiatrique est actuellement limitée. Les approches translationnelles inversées, l’utilisation d'autres -omiques et l’inclusion d'endophénotypes pédiatriques/DCPC dans les biobanques à grande échelle peuvent être des solutions possibles. La durée de la vulnérabilité développementale et des changements génomiques longitudinaux aprés la chirurgie justifie des recherches plus approfondies. L'émergence de stratégies de précision prometteuses basées sur lé'dition de génes et la programmation épigénétique pour la prise en charge de la douleur font valoir la nécessité de poursuivre les recherches sur la génomique pédiatrique liée à la DCPC.

## Introduction

Chronic postsurgical pain (CPSP) has recently been recognized as an entity in the *International Classification of Diseases*, 11th Revision.^1^ It is being increasingly studied in pediatric cohorts where the incidence is reported as 14.5% to 38%.^1,2^ Importantly, up to 33% of preterm babies require surgery, and a higher proportion undergo painful procedures in the neonatal intensive care unit (NICU). Major surgery within the first 3 months of life has been associated with increased pain sensitivity and analgesic requirements with subsequent surgeries compared with infants with no prior surgery, and time spent in the NICU has been linked with increased nociceptive sensitivity in school-aged children, possibly due to repeated painful stimuli received as neonates.^3,4^ With a high likelihood of hypersensitivity later in life,^5^ the reported incidence of CPSP in children is likely just the tip of the iceberg for this phenomenon and is only likely to increase in the future.^6^ The presence of preoperative pain and acute postoperative pain intensity (poorly controlled pain in the immediate and subacute periods) have been identified as risk factors for the development of CPSP,^7,8^ so much of the early research in this field focused on understanding the mechanisms underlying acute pain after surgery as a way of preventing the transition to CPSP. Psychosocial factors such as anxiety sensitivity,^7,9^ perioperative factors such as surgical duration,^7^ and parent–child interactions^,3 2,10^ have been shown to have both positive and negative influences on CPSP development in children.^11^ These factors have ~72% accuracy in explaining 16% of interindividual CPSP susceptibility variability in children undergoing spine fusion.^7^ The heritability of chronic pain susceptibility is estimated at ~50%^12–14^ based on family and twin studies, with genetic effects accounting for 12% to 60% response variability to experimental pain^15^ and chronic pain conditions.^16–19^ This points to a genetic contribution to individual differences in chronic pain risk and/or severity, but the specific genetic architecture of CPSP remains incompletely understood. In addition, shared environmental factors are responsible for 7% to 10% variance in chronic pain development.^16^ Similar to other chronic pain conditions, there is increasing evidence to show that genetic factors linked to CPSP risk^13,20,21^ intricately interact with environmental factors to play a role in the transition of acute to chronic postsurgical pain.^22^ Thus, in addition to genetics, epigenetic mechanisms have been a focus of study in development and maintenance of CPSP.

Though risk factors for CPSP and its related sequelae have been identified in clinical populations, the heterogeneity of patient demographics and surgical procedures, comorbidities, varying standards of care/pain definitions, and subjectivity of pain measures after surgery add complexity to clinical research.^23^ Hence, preclinical models for CPSP are essential to understanding the pathological processes underlying CPSP and allow researchers to ask questions that could not be answered easily in the clinical setting. In this review, we discuss preclinical to clinical evidence for the role of genomics (genetics and epigenetics) in pediatric CPSP. We describe benefits and limitations of animal models used to study CPSP and discuss challenges of translational research. We also discuss epigenetic and genetic signatures in nociceptors and immune cells modulating neonatal nociceptive priming, an important concept leading to chronic pain transitions in children. We review current clinical studies in children describing genetic and epigenetic associations with CPSP and draw parallels with findings from adult genetic studies where there is a scarcity of pediatric evidence. Finally, we elaborate on integrative approaches of basic and clinical research, potential targets for novel therapeutic strategies in human subjects, and future areas of research.

To better understand the nuances of extrapolating adult findings to pediatric populations, it is important to understand the differences in physiology of the developing nociceptive system compared to adults. In adults, there is good evidence that amplification of neural signaling within the central nervous system leads to central sensitization, contributing to many prolonged chronic pain states.^24^ However, an immature neonatal brain is not just a small adult brain. During brain development, a progressive reduction of intracellular chloride in neurons leading to an associated switch in gamma amino butyric acid (GABA) polarity (excitability and generation of depolarizing potentials in immature brains to hyperpolarization and inhibition) has been confirmed in a wide range of animal species.^25,26^ Also, it has been shown that up to postnatal day 21 (P21) in the rat, the rostroventral medulla of the brainstem exclusively facilitates spinal pain transmission but that after this age (P28 to adult), the influence of the rostroventral medulla shifts to biphasic facilitation and inhibition,^27^ and this switch may be mediated by mu-opioid receptor pathways.^28^ Although sensory neurons, including nociceptors, display age-related changes in functional makeup during early development,^5,29^ nociceptors can be functional by the 20th week of gestation. The peripheral sensory neurons in the dorsal root ganglia (DRG) overall appear to be fully developed by early childhood as external stimuli continue to shape their maturation.^29^ However, interneuronal communications in the spinal cord are still developing at early ages. Hence, the premature newborn brain can poorly distinguish noxious and innocuous stimulation. Importantly, nociceptive reflexes and microglial reactions are strong at an early age, and repeated nociceptive stimuli (depending on age of initial insult) lead to irreversible changes that persist into adulthood, causing hyperalgesia, increasing risk for developing chronic pain, enhanced cortical activity to noxious stimulation, and considerable alterations in somatosensory and pain processing.^30,31^ We believe this brief prelude will highlight and provide a context for genomic evidence presented for pediatric CPSP as well as help readers understand relevant pediatric connections where adult findings are described in the article.^27,28^

## Preclinical Models Relevant to Pediatric CPSP

**Figure 1.**
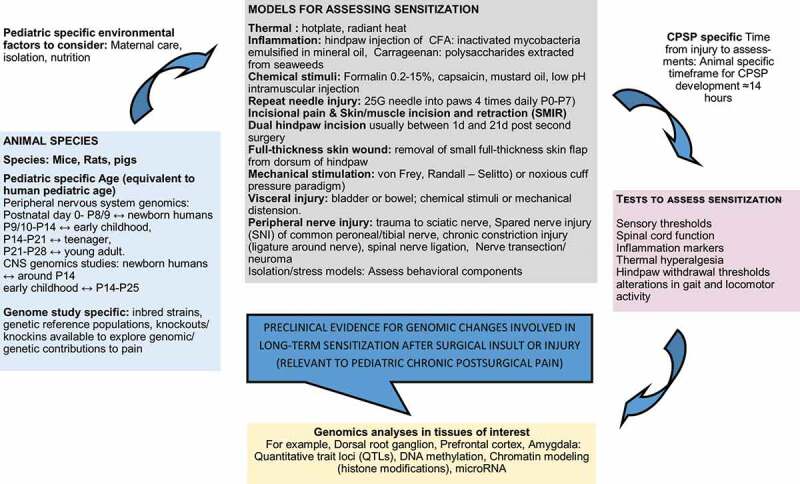
Diagrammatic representation of preclinical pain models, tests, and analyses used in genomic studies with relevance to pediatric chronic postsurgical pain phenotypes.

Though there are many models that exist to study genomic/genetic/epigenetic factors contributing to postsurgical pain, only a subset of these are commonly applied to CPSP explicitly, and even fewer have been leveraged to investigate pediatric CPSP specifically. Refer to Figure 1 for a brief overview of preclinical surgical models relevant to pediatric CPSP genomic investigations. Detailed reviews on pain assessments in experimental models of neonatal and pediatric pain from early life sensitization have been previously published.^6,32^ Rodents (primarily mice and rats) are the most common animal model for pain genetics research, but there are several caveats to using these models for the study of pediatric CPSP. Mice are born at an earlier point of maturation compared with full-term birth in a human, equating roughly to the second postnatal week in rodents.^33^ In addition, general maturational rates are not linearly correlated between rodents and humans; mice mature at ~150 times the rate of humans in the first month, and this ratio decreases to 25:1 after 6 months of age. As a result, if pediatric CPSP is defined clinically as pain lasting >3 months, this would correspond to ~14 hours in the first month of life for a mouse,^34^ but most studies have used a much more protracted time frame (on the order of days to weeks depending on the specific surgical model) for measuring hypersensitivity after surgery/injury in adolescent mouse models, even in this early period of accelerated development. Quantification of chronic pain severity in these models is often accomplished using pain-eliciting stimuli^35–41^ where severity of pain is associated with the degree of hypersensitivity exhibited or through pain measures such as alterations in gait and locomotor activity that more effectively mimic movement-evoked pain as seen during surgical recovery.^42^

Preclinical models have proven useful for studying the effectiveness of common therapeutics for acute pain due to injury or inflammation (e.g., morphine, gabapentin, etoricoxib, celecoxib, indomethacin, naproxen) in the prevention and treatment of CPSP.^43,44^ However, pediatric studies are needed to allow translation of findings in adult to pediatric applications. For example, the development of anti-calcitonin gene-related peptide antibodies for the treatment of migraines in adults is currently being followed up by pediatric pharmacokinetic studies^45^ to determine whether dosing schedules based on weight or body surface area or hybrid models are optimal, because younger children have faster clearance and lower plasma concentrations when dosed based on weight and age.^46^ In addition, safety, potential immunogenicity, and effects on pediatric physiology may be very different from those for adults. Despite the unknowns, which can only be resolved by long-term safety and efficacy trials in children, recommendations for its use in children with refractory migraine have been put forth by experts,^47^ showing promise for potential translational success on CPSP therapeutics in children.^48^ Nevertheless, animal models have limitations for translation. Behavioral responses to pain differ widely, with no clear-cut patterns between rats from the same strain purchased from different suppliers and different strains of mice, influencing both genetic association and interventional findings.^49,50^ Because pain is a biopsychosocial phenomenon, it is not amenable to assess the wholesome nature of this phenotype in animals, although certain models (acetic acid [0.9%] writhing test and manipulating social partner) have been used to simulate social environments. Despite interesting targets for therapies in animals (for example, neurokinin 1 antagonists), translation to human domains has been elusive.^51^ Nevertheless, pain memory, an important risk predictor in pediatric acute to chronic pain transitions,^52^ has been observed in animal studies showing long-term sensitivity following injury.^53^ In addition, reverse translation, by first identifying variants associated with CPSP in human studies followed by mechanistic investigations in animal models, is suggested as a potentially improved approach to bridge the gap between benchside research and bedside applications.^54,55^

## Preclinical Genetic Evidence in Chronic Postsurgical Pain

Findings from unbiased genome-wide approaches in animal models that recapitulate the tissue damage/injury aspects of surgery can provide insight into potential pediatric CPSP-relevant candidate genes. One such method, quantitative trait locus (QTL) mapping, has successfully identified multiple genomic loci in rodents where genotype is correlated with variation in the susceptibility to chronic pain; though these studies have not been conducted in juvenile mice, the data can be used to generate hypotheses for subsequent testing in pediatric CPSP. We could find no genomewide analysis conducted in animal models with the goal of identifying potential risk alleles or variants for pediatric-specific CPSP. However, two relevant QTLs, *pain1* (mouse chromosome 15)^56^ and *pain2* (rat chromosome 2),^57^ conducted in adult rodents have identified genomic loci associated with chronic pain in the neuroma model of sciatic nerve transection that shares similarities to surgical and traumatic amputations. *Pain1* contains 155 genes, but using whole genome microarray expression analysis and bioinformatics, a single high-priority candidate, *Cacng2*, was identified. A *Cacng2* hypomorphic mutant mouse confirmed the gene’s functional role in chronic pain susceptibility, and subsequent translational studies revealed human *CACNG2* single-nucleotide polymorphisms (SNPs) predicted risk for CPSP in adult women.^57^ A similar approach was used to identify and confirm a role for purinergic receptor P2rx7^58^ in susceptibility for nerve injury–induced mechanical hypersensitivity. This provides strong support for uncovering the genetic basis for CPSP with genome-wide linkage mapping or similar preclinical tools. Animal models offer the opportunity to examine the role of specific candidate genes identified in clinical populations.^13^ A direct example of this approach for CPSP comes from work by Tian et al.,^59^ who sequenced 638 SNPs associated with 54 candidate pain-related genes in patients with CPSP and, as a result, identified brain-derived neurotrophic factor as a high-priority candidate gene. Knock-in mice harboring this specific brain-derived neurotrophic factor mutation were found to have decreased mechanical sensitivity corresponding to their human cohorts, indicating lower risk for CPSP. Though these methods are available and reliable, their application to *pediatric* CPSP has lagged behind their application to other forms of chronic pain.

Even with the paucity of unbiased whole-genome approaches being used in preclinical models of pediatric CPSP, future animal studies are critical to disentangling the individual differences involved in CPSP risk by offering (1) an enhanced level of precision for identifying the location, timing, and specific mechanisms by which individual genomic differences (genetic, or epigenetic) contribute to the pathology underlying pediatric CPSP and (2) a substrate for discovery of alternative therapeutics for treatment and prevention of pediatric CPSP. The fundamental genetic/epigenetic contributions to pediatric CPSP have yet to be identified, but the systematic control over environmental parameters in animal studies makes them ideal for this type of inquiry, and eventually these methods could be used to model the multiple clinical factors that likely contribute to CPSP in the clinical setting, including insufficient postoperative pain control,^60^ presence of drains, postoperative infection,^61^ and postponing the use of antineuropathic medication.^62–64^

## Preclinical Evidence for Epigenetic Mechanisms in Chronic Postsurgical Pain

Epigenetic modifications alter gene expression without altering the DNA sequence through processes including DNA methylation,^65^ chromatin remodeling through histone modifications (methylation and acetylation), and noncoding RNAs (e.g., miRNAs)^66–72^ that regulate gene expression.^67,73^ Prior work illustrates a number of specific alterations in epigenetic status induced in models of surgery-like injury, but, again, the application of these findings to the pediatric-equivalent in rodents is extremely limited. Nerve injury has been shown to induce global DNA *hypo*methylation in the DRG but global *hyper*methylation in the spinal cord and prefrontal cortex, pointing to the importance of tissue-specific changes in interpretation. To this end, Denk et al. previously proposed persistent, postinjury epigenetic alterations at microglial enhancers in spinal mechanisms underlying pain chronicity.^74^ Chronic painful neuropathy induces persistent DNA hypomethylation in the prefrontal cortex and amygdala^68^ with a concomitant increase in of S*ynaptotagmin II* (*syt2*) expression, which plays a role in synaptic vesicle docking and as a calcium sensor for fast neurotransmitter release.^75^ These findings specifically point to an anatomical and epigenetic substrate for the emergence of psychological comorbidities of chronic pain, but their impact in the context of the immature brain in pediatric patients is unclear.^68,76^ Similarly, peripheral inflammation induces active DNA demethylation of the *cbs* gene promoter region in primary sensory afferents, resulting in increased production of hydrogen sulfide and increased pain.^77,78^ Other reports implicate differential methylation and hypoxia-inducible factor 1 signaling pathway gene expression in neuropathic pain severity in both rodent models and breast cancer survivors.^79^ Relevant to CPSP, increased methylation of the mu- and kappa-opioid receptor promoters in DRG neurons following nerve injury provides a potential mechanism underlying the opioid resistance of neuropathic pain in preclinical and clinical populations.^80^

Histone deacetylase (HDAC) levels increase in the spinal cord as a result of peripheral inflammation and nerve injury, suggesting a role in pain persistence and/or chronicity.^77,81^ In fact, inhibiting spinal HDAC activity attenuates nerve injury–induced hypersensitivity.^82^ To this end, neuropathic pain reduces histone methylation, resulting in persistent dysregulation of the immune response to nerve injury.^83^ Though these data are not specific to pediatric CPSP, they do shed light on potential therapeutic targets for the prevention of CPSP given the involvement of both inflammation and tissue injury in most surgical procedures and the sensitivity of the epigenome to transient alterations during the pediatric developmental stage.

In one of the only specific investigations of CPSP, a rat model of lingual nerve injury, a common occurrence during routine oral surgery or facial trauma/reconstruction, lingual nerve expression levels of multiple miRNAs predicted to regulate inflammatory and pain-related pathway genes were correlated with pain behavior. The relationships held true when miRNA expression in lingual neuromas was correlated with patient pain ratings.^84^ miRNAs may contribute to alterations in sensory neuron excitability through their regulation of sodium channel (Na_v_) expression levels. The miRNAs miR-96^85^ and miR-7a^86^ exert regulatory control over Nav1.3 following nerve injury; the specific deletion of the miRNA processing enzyme Dicer in the DRG reduces expression of Nav1.7, 1.8, and 1.9 channels and attenuates inflammatory pain behaviors.^87^ Relevant to the use of opioids for postoperative pain, the Let-7 group of miRNAs has been implicated in the development of morphine tolerance, offering a potential mechanism by which miRNAs could play a role in the opioid resistance of CPSP that affects both adult and pediatric patients.^88^ Further functional studies examining the tissue-specific roles of all epigenetic modifications in the emergence of CPSP are needed, and this is particularly true for their role in pediatric CPSP.

## Neonatal Nociceptive Priming: Epigenetic and Genetic Signatures in Nociceptors

A critical concern with children experiencing early life pain is how development of the nociceptive system is affected. Clinical and rodent data demonstrate that there are discrete time periods in which an aversive stimulus, such as an injury, results in altered development and long-lasting changes to the somatosensory system.^4,89^ Individuals who experience early life pain are at an increased risk of complications after an injury later in life, a phenomenon called *neonatal nociceptive priming*.^90,91^ Importantly, these effects are clinically relevant to neonates who undergo painful stimuli within the NICU. Hypersensitivity to tissue damage resulting from repeated heel sticks/procedures during clinical neonatal intensive care can persist long-term.^6^ The specific role of different sensory neurons has been extensively studied in adult pain, but the role of specific sensory neurons in the onset of neonatal pain is not clearly understood.^92,93^ Reports have indicated that age is a key factor that modulates pain after peripheral nerve injury.^93,94^

The normal development of somatosensory and pain processing is dependent on the sensory information from skin, muscle, and joints, which relay information to the spinal cord during the first few postnatal weeks.^92,95,96^ Primary sensory neurons of the DRG that respond to touch, pain, temperature, itch, etc.,^97^ are chemically and functionally heterogenous.^97,98^ DRGs undergo many phenotypic changes during early postnatal development that are regulated by target-derived neurotropic factors (NTs).^96,97,99,100^ These factors exhibit temporal influence on developing primary afferents and alter the responses of neonatal sensory neurons to peripheral stimuli in response to injury.^29,93,96^ A functional switch from mechanically sensitive, thermally insensitive C-fibers to polymodal C-fibers during postnatal development^29^ coincides with the previously described neurochemical switch in growth factor responsiveness.^101^ Thus, peripheral injury prior to or after this critical period results in distinct sensitization patterns in the DRG neurons. Unique pharmacological and behavioral responses to injuries exist between developing and adult subjects, and this is observed in both patients and animal models.^31,92^ Potential neonatal-specific analgesic properties and mechanisms of nociceptive signaling also lend credence to the presence of a “primed” nociceptive system that enhances the response to re-injury later in life.^92,102^

The genetic landscape of human and animals models is known to play important roles in the onset and perpetuation of chronic pain stemming from early life injury.^12,13,67,103^ Recent evidence demonstrates that neonatal mechanisms of nociception are distinct from those of adulta,^29,104,105^ and early life injury has been shown to change patient sensitivity to peripheral stimuli in adulthood.^93,106^ When considering alterations in development, previous data indicate differences in chromatin accessibility between early stages of life and later developmental time points across different cell types.^107^ Cellular activity can alter epigenetic signatures, and immunological data suggest that innate immune cells use the epigenome as a form of cellular memory.^108,109^ Further, animal models have identified alterations in neuronal function and differentiation through epigenetic modifications.^110,111^ However, the effect of injury and the direct impact this has on the nociceptive system is unknown. The complex interactions and genetic variation between patients such as SNPs^12,13,67,112^ and epigenetic modifications have gained attention in the onset of early pain.^12,66,67^ However, the cell types, systems, and localization of neonatal nociceptive priming remain pertinent questions.^113^ It will be necessary to determine the underlying factors that contribute to the unique vulnerability of the neonate, especially at the level of the sensory neuron in the form of a cellular “memory.” Hence, the definitive classification of primary sensory neurons at the single cell level over time under normal and pathological conditions will help identify genes involved in sensory neuron function and their role in neonatal priming. We are working to determine how subpopulations of sensory neurons are altered through development and the impact that early life injury has on the different subtypes at the functional and epigenetic levels. This type of analysis will be of critical importance to determine whether early life surgical incision drives chromatin accessibility modifications that contribute to neonatal nociceptive priming.

### Neonatal Nociceptive Priming: Role of Macrophages

It is clear that within the spinal cord, both dorsal horn circuitry and microglia, the macrophages of the central nervous system, are critical for neonatal nociceptive priming.^91,114,115^ However, evidence suggests that peripheral input through the primary afferents is also necessary for this.^105,116^ Nociceptive input is transmitted via primary afferent nociceptors and is modulated by the immune system.^117^ Importantly, macrophages undergo robust developmental changes in early life and experience a critical period that overlaps with the vulnerable period of the somatosensory system.^118–121^ Following infection or injury, a number of biological factors are released to the affected tissue, and macrophages begin to populate the area.^117^ The pro- or anti-inflammatory profile and presence of macrophages have been linked to patient and animal outcomes following surgical injury during development.^122,123^ Together, these data suggest that activated macrophages in the neonate are unique and important in acute nociception as well as a long-term predisposition to chronic pain.

A unique feature of the peripheral immune system is its known ability to retain cellular memory. Though this “memory” is best attributed in the adaptive immune response, the innate immune system can also establish memory. In animals lacking an adaptive immune system, macrophages recognize pathogens to which they were previously exposed^109^ through the unique pro- or anti-inflammatory microenvironment, signaling cascades, and epigenetic modifications.^108,124^ The microenvironment in the tissue creates a signature of cytokines, chemokines, and growth factors known as pathogen-associated molecular patterns and/or damage associated molecular patterns.^125^ These are recognized by innate immune cells, including dendritic cells, natural killer cells, and macrophages, by pattern recognition receptors. The activation of these “lock and key” signals to receptors on macrophages induces intracellular signaling cascades altering transcription factors and the epigenetic landscape, which contributes to the formation of the immune memory.

Other molecules that directly alter the genome, such as HDACs, also drive epigenetic changes by modulating specific promotor regions to induce or inhibit pro- or anti-inflammatory responses from effector cells. Chromatin alterations include poised chromatin (e.g., H3K4me3 and H3K27me3), heterochromatin (e.g., H3K27me3 only), and active chromatin (e.g., H3K27ac and/or H3K4me3 only) or repressive chromatin (H3K9me2).^126,127^ Each of these modifications can induce long-lasting changes in gene expression^128^ and are specific to the pattern of stimulation.^109^ It is important to note that the epigenome in early life is unique in macrophages,^129^ necessary for tissue resident development,^130^ and is required for monocyte transition into macrophages.^131^

Macrophages have been found to become either “trained” or “tolerant” to certain stimuli. If trained, macrophages that are restimulated with a factor that they had previously encountered will display an increased pro-inflammatory response. Opposing this, macrophages that become tolerant to repeat stimuli have a reduced inflammatory response. The difference between these has been traced to differential epigenetic regulation on the promotors of effector genes. For example, stimulation to trained immunity can result in persistent active chromatin marks, whereas stimulation to tolerant immunity results in repressive marks.^124,132^ In either case, after cessation of the cellular response following the “first hit,” the cell resumes similar activity. It is not until a “second hit” that the priming effect within the cell is observed.^132^ The factors that regulate this and the epigenetic landscape following different stimulations have been recently reviewed by Fanucchi et al.^133^ Although it is clear that macrophages have distinct responses after restimulation, the timescale, developmental vulnerability, and effect after injury are less explored and warrant further investigation.

After a tissue breaking injury, including surgery, a number of biological and cellular systems initiate the injury response and facilitate repair of the damage.^134^ The peripheral immune and nervous systems work together by sending signals to one another in a bidirectional pattern and to alter the local microenvironment.^135,136^ Previous data and our recent unpublished data indicate that the microenvironment after a neonatal surgical injury may be unique from that of the adult.^105,129^ Our work further demonstrates that macrophages are necessary for animals to display acute pain-like behaviors after an early life incision as well as chronic pain-like behaviors after a repeat injury later in life. The mechanisms that underlie macrophage involvement in maintaining memory of early life surgical injury may be similar to the mechanisms that underlie macrophage involvement after an infection and may be controlled by the epigenome. The unique properties of the neonatal macrophage and immune response may contribute to the vulnerable periods for both the peripheral immune system and nervous system. Because neonatal macrophages display a unique epigenetic landscape compared to adults,^129^ these data indicate that pediatric surgery may drive macrophage modifications that are long-lasting and affect injury outcomes later in life.

## Clinical Studies of CPSP in the Pediatric Population

**Figure 2.**
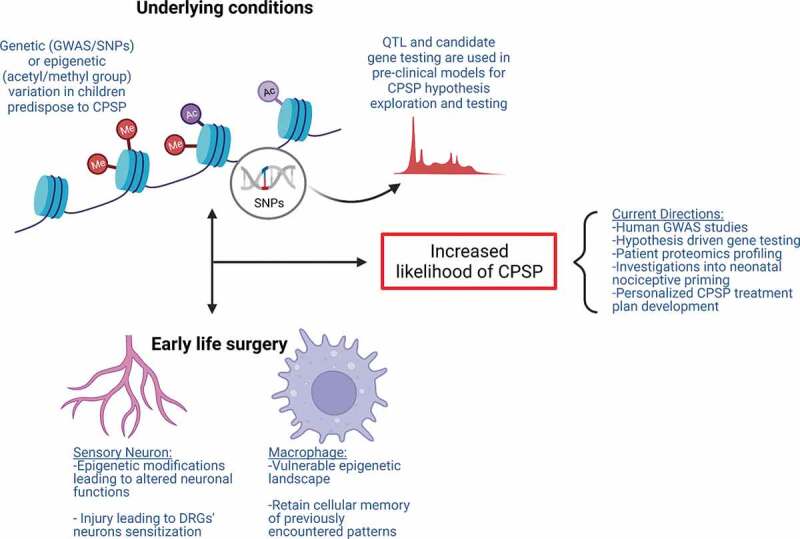
Mechanisms contributing to increased susceptibility to CPSP. Underlying molecular mechanisms comprising genetic variations (i.e., SNPs) and epigenetic modifications (i.e., DNA or histone methylation and acetylation and miRNAs) contribute to individual differences in tissue-specific gene and protein expression in clinical association studies. Gene and protein expression differences can account for increased risk for altered neuronal excitability and sensitization. Alternative mechanisms involved in nociceptive priming are instigated following early life surgery. Tissue injury incites tissue-specific alterations (i.e., epigenetic modifications, gene expression changes) in cell types including sensory neurons and macrophages, which may be important in the formation and maintenance of neonatal nociceptive priming. Underlying conditions and early life surgery can independently contribute to increased susceptibility to CPSP and even act in a feedforward loop together, exacerbating CPSP.

Pediatric clinical cohorts in CPSP genetic association studies are mostly small samples and thus findings need further scaling and validation. That said, the findings are mostly aligned with prior basic science knowledge, and novel systems biology–based approaches have been used to overcome size limitations. A schematic representation of the mechanisms involved in postinjury nociceptive priming from preclinical evidence is presented in Figure 2.

### Genetic Association Studies with CPSP

Recent systematic reviews describe CPSP–genetics associations.^137–139^ In a comprehensive review of 21 CPSP gene association studies by Chidambaran et al., only one study included pediatric subjects (14–35 years) but the number of adolescents recruited was not stated.^140^ They conducted a meta-analysis including six variants of five genes (CO*MT*: rs4680 and rs6269, mu-1-opioid receptor/*OPRM1*: rs1799971, GTP cyclohydrolase 1/*GCH1*: rs3783641, potassium voltage-gated channel modifier subfamily S member 1/*KCNS1*: rs734784, tumor necrosis factor/*TNFA*: rs1800629),^141–149^ but only rs734784 (A > G) of *KCNS1* was found to marginally increase CPSP risk (additive genetic model; odds ratio = 1.511; 95% confidence interval [1–2.284]; *P* = 0.050). In another study, *COMT* rs4860 and μ-opioid receptor rs1799971 were not found to contribute to CPSP development after cesarean delivery.^150^ Warner et al. conducted a GWAS meta-analysis and reported that a variant in protein kinase C alpha gene (*PRKCA*) gene was associated with neuropathic pain following total knee replacement,^151^ but this was not replicated in other studies. Another GWAS **Genome wide association studies** in females posthysterectomy showed that rs118184265 at *NAV3* was associated with CPSP in the replication cohort. Loci at cAMP response element-binding protein (CREB)‐regulated transcription co‐activator 3 gene (*CRTC3*) (rs117119665) associated with CREB-dependent transcription of genes and IQ motif containing GTPase‐activating protein 1 (*IQGAP1*) (rs1145324) involved in immune signaling were significantly associated with CPSP in a meta‐analysis in both the discovery and replication cohort.^152^ However, the study was underpowered due to the small size of the discovery cohort. Heterogeneity in surgical cohorts, population structure, outcome definitions, unbalanced sex ratios, and the small cohort sizes are likely responsible for lack of consistent and replicable findings. For example, *KCNS1* variant rs734784 A > G (Ile48Val) was associated with higher pain scores in patients with disc herniation and lumbar back pain, phantom limb and stump pain in amputees, preoperative sciatica pain, and experimental pain sensitivity^148^ but not with long-term pain after breast cancer surgery,^153^ raising the possibility that this variant might increase risk for neuropathic CPSP but not nonneuropathic pain.^154^ Table 1 summarizes the role of genes involved in variant CPSP association studies from the literature. It is unclear whether these findings will be replicated in pediatric cohorts. Although acute postsurgical pain and analgesic requirements are important predictors of CPSP in children^7^ and genetic influences on both of these factors may play a role in CPSP, this is beyond the scope of this focused review on pediatric CPSP genomics. Detailed reviews on these aspects have been previously published.^155–157^

**Table 1.** Literature-curated list of genes/variants associated with chronic postsurgical pain and their function

Gene	Function^a^	Risk variant	Protective variant	Reference
Catechol-O-methyl transferase (*COMT*)	Neurotransmitter degradation—metabolism of noradrenaline, adrenaline, and dopamine; regulates pain perception, cognitive function, and mood	rs6269 T, rs4633, rs4680 ATC Haplotype: rs4646312T>C, rs165722T>C, rs6269A>G, rs4633T>C, rs4818C>G, rs4680A>G, rs6269G, rs4633T rs4680 A High pain sensitivity haplotype rs4680 A, novel mutations c.382 C > G, c.383 G > C, p.(Arg128Ala)	rs4633 T; rs6269 G (Belfer) Haplotype L (rs6269, rs4633, rs4818, rs4680 A_C_C_G (Rut) rs6269, rs4633, rs4818 G_C_G (Belfer)	Rut 2014^158^ Belfer 2015^137^ Dimova 2015^130^ Dharaniprasad 2020^146^ Knisely 2018^147^
Calcium voltage-gated channel subunit gamma 2 (*CACNG2*)	Brain-specific transmembrane protein that modulates the trafficking and ion channel kinetics of glutamate AMPA receptors; subunit of neuronal voltage-gated calcium channels	A-C-C haplotype at rs4820242, rs2284015, and rs2284017		Nissenbaum 2010^148^ Bortsov 2019^159^
Potassium voltage-gated channel subfamily D member 2 (*KCND2*) subfamily J member 3 (*KCNJ3*) subfamily J member 6 (*KCNJ6*) Potassium voltage-gated channel modifier subfamily S member 1 (*KCNS1*) Potassium two-pore domain channel subfamily K member 3 (*KCNK3*) Potassium two-pore domain channel subfamily K member 9 (*KCNK9*)	Voltage-gated potassium channel subunits mediate transmembrane potassium transport primarily in the brain (*KCND2*) GPCR-regulated potassium channel G protein–gated inward rectifier potassium channel (*KCNJ3*) Potassium channel that regulates insulin secretion by glucose and neurotransmitters (*KCNJ6*) Modulates the delayed rectifier voltage-gated potassium channel activation and deactivation rates of KCNB1 and KCNB2 (*KCNS1*) pH-dependent, potassium channel rectifier (*KCNK3/KCNK9*)	*KCNJ3* rs6435329 T, rs11895478 T, rs3106653 C, rs3111006 T, rs12471193 G, rs7574878 G, rs12995382 C *KCND2* rs802340 T *KCNJ6*: rs2835925 C/G, HapE2, HapE7 *KCNK3*: rs1662988 T, rs7584568 A, HapB1, HapB4 *KCNK9* rs2014712 T *KCNS1* rs734784 G, rs13043825	*KCND2* rs17376373 *KCNJ3* HapA2 rs3111020–rs11895478 G_A; Hap B1, HapB4, HapC5	Langford 2015^160^ Costigan 2010^138^
Sodium channel alpha subunit gene (*SCN9A*)	Voltage-gated sodium channel Nav1.7 subunit	rs16851799 TT		Yeo 2020^149^
Major histocompatibility complex, class II, DQ beta 1 (*HLA-DQB1*) Class II, DR beta 1 (*HLA-DRB1*) Interleukin genes (*IL*), gamma interferon (IFNG1), tumor necrosis factor alpha (TNF alpha) C-C motif chemokine ligand 2 (CCL2) C-X3-C motif chemokine ligand 1 (*CX3CL1*)	Presentation of foreign antigens to the immune system Cytokine produced by activated macrophages (*HLA*) Pro-inflammatory cytokine (*IL1A, IL4*) Multifunctional proinflammatory cytokine mainly secreted by macrophages (TNF-alpha) Anti-inflammatory cytokine (IL-10, IL-13) Glial cell–neuron interaction (*CCL2*) Chemokine induced by primary proinflammatory signals (*CX3CL1*)	HLA-DRB1*4 *HLA-DQB1* 03:02 *DQB1* 03:02—DRB1*04 haplotype *IFNG1* rs2069727 G, rs2069718 T, HapA5 *IL1A* rs1800587 *IL1R1* rs3917332 T *IL1R2* rs11674595 C *IL4* rs2243248 *IL10* rs3024498 G, rs1878672 C, rs3024491 G, HapA8 *IL13* rs1881457 C, rs1800925 T, rs1295686, A rs20541 T, HapA1 TNF-alpha rs1800629 GG	*CCL2* rs4586 T allele *CX3CL1* rs614230C allele	Dominguez 2013^150^ Kalliomaki 2016^134^ Dimova 2015^130^ Ma 2019^151^
Nuclear factor-kappa-B proteins (*NFkB1A*)	Inhibits the activity of NF-kappa-B complexes	rs8904 T rs4648141 A		Montes 2015^133^ Stephens 2014^152^
Calcitonin-related polypeptide alpha (CALCA, α-CGRP)	Neuropeptide—mediator of neurogenic inflammation	rs3781719C allele		Ma 2019^151^
Cadherin 18 (*CDH18*) Methionine adenosyltransferase 2B (*MAT 2B*) Glycerol-3-phosphate dehydrogenase 2 (*GPD2*) Forkhead Box L1 (*FOXL1*)	Calcium-dependent cell adhesion protein (*CDH18*) Regulatory subunit of S-adenosylmethionine synthetase 2, an enzyme that catalyzes the formation of S-adenosylmethionine from methionine and ATP (*MAT2B*) Transcription factor (*FOXL1*)	*CDH18 rs4866176 A* *TG rs1133076 A* *MAT2B rs7734804 A* *GPD2 rs298235 A* *FOXL1 rs12596162 A*		Kalliomaki 2016^134^
GTP cyclohydrolase 1 (*GCH1*)	Rate-limiting enzyme for tetrahydrofolate biosynthesis	rs8007267 T rs3783641, *rs4411417 C* rs3783641, rs8007267 T_C	rs8007267, rs3783461, rs8007201, rs4411417, rs752688 CAGCT rs998259 T	Hegarty 2012^135^ Montes 2015^133^ Belfer 2015^137^ Tegeder 2006^153^ Kim 2010^154^
Protein kinase C, alpha (*PRKCA*)	Neuroendocrine receptor interactions	rs887797 A		Warner 2017^141^
Purine receptor signaling (*P2X7R)*	Receptor for ATP, acts as a ligand-gated ion channel mediating ATP-dependent lysis	rs208294 A rs208294–rs208296–rs7958311 ACG haplotype	rs208296 T rs7958311 A rs208294–rs208296–rs7958311 GTA haplotype	Sorge 2012^50^
Dopamine receptor (*DRD2*)	Adenylate cyclase inhibiting G protein–coupled receptor superfamily, expressed in basal ganglia	rs4648317T		Montes 2015^113^
Cholinergic receptor nicotinic alpha 6 subunit (*CHRNA6*)	Cholinergic receptor activation of which opens ion-conducting channel	rs7828365 TT		Weiskopf 2015^155^
CREB‐regulated transcription co‐activator 3 gene (*CRTC3*)	CREB-dependent transcription of genes	rs117119665		Van Reij 2020^142^
IQ motif containing GTPase‐activating protein 1 (*IQGAP1*)	Cytoskeleton regulation, immune signaling, and cell motility	rs1145324		Van Reij 2020^142^
ATP-binding cassette (*ABCB1*)	ATP-dependent drug efflux pump	rs1045642 T		Sia 2013^156^
Brain-derived neurotrophic factor (BDNF)	Regulator of synaptic transmission and synaptic plasticity		allele A of rs6265	Tian 2018^157^
Ataxin 1 (*ATXN1*)	Chromatin-binding factor	rs179997 A		Montes 2015^133^
Growth differentiation factor 5 (GDF5)	Regulates growth of neuronal axons and dendrites and plays a role in the inflammatory response and tissue damage	rs143384 in 5ʹ UTR Haplotypes AGG and GGG in the LD block rs143384–rs224335–rs739329		Yan 2021^158^
5-Hydroxytryptamine receptor 1A (*5HTR1A*); 5-hydroxytryptamine receptor 2A (*HTR2A*); 5-hydroxytryptamine receptor 3A (*HTR3A*)	Receptors for neurotransmitter serotonin	*5HTR1A* rs6295 G Moderate pain class: *HTR2A* rs2296972	mild pain class: *HTR3A* rs10160548	Lebe 2013^159^ Knisely 2018^147^
SLC family 6 member 2-noradrenaline transporter (*SLC6A2*) SLC family 6 member 3-noradrenaline transporter (*SLC6A3*)	Amine transporter (*SLC6A2*)—terminates action of noradrenaline by sodium-dependent reuptake into presynaptic terminals Dopamine transporter (*SLC6A3*)	Moderate pain class: *SLC6A2* rs17841327, and *SLC6A3* rs403636 *SLC6A2* HapD01 *SLC6A3* rs464049	*SLC6A2* rs1566652	Knisely 2018^147^
Beta-2-adrenergic receptor (*ADRB2*) Beta adrenergic receptor kinase 2 (*ADRBK2*)	Adrenergic signaling		*ADRB2* rs2400707 *ADRBK2* HapA04	Knisely 2018^147^
Tryptophan hydroxylase 2 (*TPH2*)	Catalyzes the first and rate-limiting step in the biosynthesis of serotonin		rs11179000	Knisely 2018^147^
Serine peptidase Cathepsin G (*CTSG*)	Serine protease with trypsin- and chymotrypsin-like specificity. Cleaves complement C3		rs2070697 AA; rs2236742 A	Liu 2015^161–173^

AMPA = alpha-amino-3-hydroxy-5-methyl-4-isoxazole propionic acid; ATP = adenosine triphosphate.

Given difficulties in developing large genetic data banks with well-characterized CPSP phenotypes in children, leveraging systems biology may offer an alternative strategy to overcome sample size limitations.^174^ Integrating genetic-level data with biologic processes can generate prioritized candidate gene lists. Chidambaran et al. demonstrated the utility of functional annotation–based prioritization and enrichment approaches to identify novel genes and unique/shared biological processes in acute and chronic postoperative pain.^175^ Certain molecular mechanisms were elucidated to be common to acute and CPSP (e.g., CREB phosphorylation, ion channels, N-methyl-d-aspartate). Certain other genetic processes played a role in CPSP but not acute pain. These included immune/inflammatory (Toll-like receptor signaling, interferon gamma signaling, cytokines, mitogen-activated protein kinase/extracellular signal–regulated protein kinase signaling) and neurotransmitter-involved processes (purinergic, oxytocin, GABA, glutaminergic, catecholaminergic, dopaminergic). Despite the findings mostly being in adult studies, some of the pathways may be pertinent to pediatric populations, based on clinical and preclinical evidence. Several genes are common to immune, dopaminergic, serotoninergic, and catecholamine pathways (described in Table 1). The latter three are also known to be involved in psychological disorders^176^ implicated in the chronification of pain in children. For example, genes involved in dopaminergic neurotransmission (catechol-O-methyl transferase [*COMT*], GTP cyclohydrolase 1 [*GCH1*], and dopamine receptor [*DRD2*]) have different mechanisms.^177^ *GCH1* is involved in the production of BH4, a key molecule in the synthesis of dopamine, and variants (rs841) decrease *GCH1* expression and are generally protective in chronic pain.^178^ *COMT* is involved in degradation of dopamine and other catecholamines with key roles in chronic pain.^179^ Its variants rs4680 and rs165774 decrease its enzymatic activity, increase catecholamine availability, and alter the signaling cascade. The dopaminergic receptors (D1-like receptor [D1LR] family [includes D1 and D5 receptors, which are stimulatory] and D2-like receptor [D2LR] family [consisting of D2, D3, and D4 receptors, which are inhibitory]) have opposite effects on nociceptive transmission. Variant rs6277 located in *DRD2* decreases the stability of mRNA, thereby decreasing the expression of the D2 receptor, and increases CPSP risk.^180,181^ This pathway in modulation of nociception after surgery thus presents excellent targets for prevention and treatment of CPSP.^182–184^

Because single variants account only for small effect sizes and different pathways play concomitant roles in CPSP development, one must consider the combined effect of several gene variants (polygenic risk) in CPSP.^17^ Polygenic risk scores (PRSs)—the sum of weighted effects of different phenotype-associated alleles—have been shown to predict several complex conditions.^185–187^ An atlas of PRS associations and putative causal relationships across the human phenome was reported, though it did not include CPSP as a phenotype.^188^ Chidambaran et al. recently combined systems biology and penalized regression techniques to determine PRS, which improved prediction of CPSP risk compared to nongenetic models.^189^ Another recent study determined a PRS that suggested significant overlap of genetics of CPSP with chronic widespread pain, rheumatoid arthritis, and sciatica (but not with chronic headache and migraine). They suggested that this overlap is potentially due to common mechanisms regulating neurological signaling (sodium channels) and inflammatory response.^190^ Interestingly, this overlap was nullified in the replication cohort when subjects were randomly reassigned. Thus, further research is needed to enumerate polygenic risk for therapeutic targeting.^191,192^

### Epigenetic Association with Clinical CPSP in Children

Epigenetic differences prior to surgery could serve as a risk factor for CPSP and tissue-specific epigenetic changes in response to a given surgery could serve as a separate risk factor.^193–203^ As evidence of epigenetic regulation of CPSP risk, offspring of mothers fed a high methyl donor diet during the perinatal period exhibit increased acute pain (mechanical allodynia following skin incision),^204–206^ highlighting the influence of DNA methylation patterns in susceptibility of injury-related pain. However, epigenetic association studies with CPSP are currently scarce^207^ and present critical research gaps, especially in pediatrics. Using C-reactive protein as a marker, epigenome-wide association studies identified hypomethylated genes contributing to inflammatory processes in CPSP.^208^ CpG methylation within tumor necrosis factor (*TNF*) gene promoter has been found to be a mechanism by which TNF alters risk for mild persistent breast pain in patients with breast cancer undergoing surgery.^153^ DNA methylation at the promoter region of the mu-opioid receptor gene (*OPRM1*) that codes for mu-opioid receptor and important in opioid pain pathways has been studied.^209^ DNA methylation at the promoter is a potent epigenetic repressor of gene transcription^210,211^ and is elevated in individuals addicted to opioids and heroin.^212,213^ In children undergoing spine fusion, blood DNA methylation in an active regulatory region of *OPRM1* gene was associated with CPSP.^214^ This region binds multiple transcription factors. It was postulated that inhibition of transcription factor binding by DNA methylation may decrease *OPRM1* gene expression, leading to decreased opioid response and increased pain responses. In contrast, another study used machine learning methods to examine a potential association between the DNA methylation of two key players of glial/opioid intersection and persistent postoperative pain 3 years after breast cancer surgery.^215^ Though their study supported a predictive utility of epigenetic testing using global DNA methylation, quantified at CpG sites located in the retrotransposon LINE1, they did not find that DNA methylation of two key genes of the glial–opioid interface (*OPRM1* and Toll-like receptor *TLR4*) contributed to the persistent pain phenotype. Chidambaran et al. investigated whole blood DNA methylation profiles using epigenome-wide association studies to identify shared, enriched genomic pathways underlying CPSP and anxiety sensitivity **Childhood Anxiety Sensitivity Index** (CASI), recognized to increase CPSP risk.^7,216^ They identified 637 CPSP-associated and 2445 CASI-associated differentially DNA methylated positions (DMPs). The DMPs associated with both phenotypes enriched GABA receptor and dopamine-DARPP32 feedback in cyclic adenosine monophosphate signaling pathways. Using bioinformatic approaches, the authors elucidated target transcription factors and downstream modifying pathways regulating genes with DMP. Aligned with the GABA findings, rodent studies have identified preoperative anxiety-induced glucocorticoid signaling downregulated Npas4 (a neuronal PAS domain protein) leading to impaired spinal GABAergic system and ultimately contributing to postoperative hyperalgesia.^217^ A schematic showing the presurgical genomic mechanisms that might increase risk for CPSP is depicted in Figure 2.

Blood DNA methylation studies may identify CPSP biomarkers. Although environmental stressor changes are expected to be similar across tissues,^218^ blood-based studies (for a neurological phenotype such as pain) could have limited mechanistic interpretation because DNA methylation is tissue specific. Although cell-free DNA (cfDNA) has not been studied in association with CPSP, reports of circulating cfDNA associations with inflammation and brain diseases such as schizophrenia^219,220^ point to potential use of cfDNA as a possible alternative to identify tissue-specific DNA methylation patterns.^221^ Functional magnetic resonance imaging and spectroscopy could also be used to identify specific brain patterns and neurotransmitters associated with CPSP epigenetic findings.^222^ However, because evidence does indicate a strong role for peripheral immune cells in CPSP development (see above), data could still play an important role in our understanding of the epigenetics of CPSP.

Postinjury and postsurgery epigenetic changes have not been studied in detail in vivo.^223^ The few cross-sectional studies cannot capture dynamic epigenetic mechanisms, making it difficult to identify direction of causality.^224^ Prospective longitudinal studies are needed to address reverse causation (epigenomes influenced by, rather than causal of, pain maintenance states). Within-subject studies will also be necessary to help control for potential confounders from associations of heritable SNPs with large DNA methylation–level differences near polymorphisms (cis effects) and associations of DNA methylation level differences with variants elsewhere in the human genome (trans effects).^225^

Niculescu identified pain-related blood gene expression biomarkers for CPSP (*MFAP3, GNG7, CNTN1, LY9, CCDC144B*, and *GBP1*), some of which are targets of existing drugs.^226^ There are plasma and cerebrospinal fluid biomarkers associated with pain,^227^ but many of these remain unexplored in relation to CPSP.

## MeQTLs: At the Intersection of Genetics and Epigenetics

Characterizing the complex relationship between genetic, epigenetic, and transcriptomic variation has the potential to increase understanding about the mechanisms underpinning CPSP phenotypes and how to influence the risk. Understanding gene–environment interactions underlying CPSP is an important area of research that is yet not well explored. One such mechanism includes methylation quantitative trait loci (meQTL), which are variants that influence DNA methylation at close or distant genomic loci. meQTLs were recently evaluated as mediators of genetic association with CPSP in a study in adolescents undergoing spine fusion.^228^ Their rationale was based on the overlap of genetic variant and DNA methylation–enriched pathways associated with CPSP that they had previously reported on. This pilot study utilized causal inference tests to report that DNA methylation at 127 cytosine–guanine loci mediated association of 470 meQTLs with CPSP. They noted that several CpG–meQTL pairs were annotated to differentially methylated regions located at PARK16 locus on Chromosome 1, where CPSP risk meQTLs were associated with decreased DNA methylation at *RAB7L1* and increased DNA methylation at *PM20D1* genes. This region has previously been implicated in dopamine processing disorders of the nervous system.

## Future Directions and Emerging Therapeutics and Interventions

Forward (bench to bedside) as well as backward (clinical to basic science) translation is needed to determine innovative targets and CPSP risk mitigation strategies. It is too early for tests based on newly discovered associations to provide stable estimates of genetic risk for CPSP. Although major findings are unlikely to be false positives, estimates based on combinations of current risk alleles need constant revision as new loci are found. In addition, CPSP may be too diverse a phenotype to have common genomic underpinnings—perhaps, study of endophenotypes and subgroups of patients having different characteristics based on biological pathways involved in the nature of pain (for example, predominantly nociceptive versus neuropathic), surgical nature (for example, musculoskeletal versus visceral), and socio-behavioral features will be a solution, as has been applied in developmental psychopathology.^229^ Furthermore, inclusion of children and CPSP as a phenotype (especially now that is a recognized *International Classification of Diseases*, 11th Revision entity)^1^ within large-scale genetic studies (for example, the UK Biobank registry)^188^ would allow genome-wide approaches to pediatric CPSP. Thus, we remain optimistic that in the future, genetics combined with other biomarkers could preoperatively stratify CPSP risk, guiding prevention and treatment. Though some gene association studies also investigated gene–gene,^142^ gene–sex,^230^ and gene–psychological factor interactions,^231^ research of such interactions, including gene–epigenetic interactions,^211^ is still in its infancy, and further research is needed to understand acute to chronic postsurgical pain transition, especially in children.

Several promising emerging therapeutics targeting genes and proteins first identified in animal models and involved in the transition from acute to chronic pain have been detailed previously.^134,193,202,232–236^ Gene editing^237^ and the development of novel chemical decoys^238,239^ that target the neurobiological substrates of chronic pain offer the potential for precision pain management strategies based on manipulating this genetic context to effectively protect patients from CPSP without the negative side effects of opioids. Though a new CPSP treatment option has been slow to emerge, understanding and targeting genes, gene expression, and the processes that regulate expression represent a logical next step in developing precision pain management for CPSP.

Epigenetic biomarkers are being developed for screening in some areas like cancer. They are also being used to develop therapeutic targets. Sun et al. found that DNA methyltranferase (DNMT) inhibitor 5-Aza-2ʹ-deoxycytidine significantly reduced incision-induced mechanical allodynia and thermal sensitivity.^240^ Although six epigenetic drugs are approved for use in the United States (many more under development), their nonspecific effects are a significant drawback (see reviews^241,242^). In addition to generalized epigenetic targeting approaches, gene-specific epigenetic targeting is becoming a possibility through recently developed genome editing technology (e.g., demethylation of specific CpGs in human cells using fusions of engineered transcription activator–like effector repeat arrays, TET1 hydroxylase catalytic domain) that can effectively target and demethylate individual genes in vitro.^243^ In addition, Cas9 systems offer novel individual gene targeted approaches.^244^ Interestingly, the beneficial effects of lifestyle modifications (e.g., exercise) on mechanical and thermal hypersensitivity after sciatic nerve injury^245^ are partially mediated by decreased HDAC activity and increased acetylation of histones in the spinal cord,^246^ pointing to the potential use of nonpharmacologic strategies targeting the epigenome in the management of CPSP.

Pharmacogenomic profiles are also being generated for individual patients in order to develop better pain management strategies.^247,248^ For example, research on the mu-opioid receptor has depicted several polymorphisms that could lead to a tailored targeting of an identified SNP.^247^ Similarly, there have been some studies using proteomics to study different types of pain,^249,250^ such as widespread musculoskeletal pain,^251^ abdominal pain,^252^ and low back pain.^253^ Modifying the existing drugs to target these proteins’ functionality may achieve the goal of treating CPSP, but proteomics profiling of pediatric populations would be a required first step to determine the utility of this strategy.

## Conclusion

There is much work to be done to understand pain-related genomics and DNA methylation changes, the crosstalk between modifiable environmental factors and pain, optimal times to intervene to prevent acute to chronic pain transitions, and identification of optimal pathways to target therapeutically. Future treatment may include epigenetically programmed drugs^254^ or simple modifications to preoperative regimens, including nutrition,^255^ activity, mindfulness, or behavioral therapy,^256–258^ to prevent persistence of pain after injury or surgery. Distinct cellular interactions must also be taken into consideration in order to enhance translational potential. Clear evidence suggests a role for both neurons and immune cells (among others) in the epigenetic regulation of CPSP. Changing bidirectional communication between neurons and immune cells is essential for proper transduction of sensory stimuli over the life span and should therefore be contemplated when developing future treatments for CPSP in children.
